# Luteolin-Mediated Inhibition of Hepatic Stellate Cell Activation via Suppression of the STAT3 Pathway

**DOI:** 10.3390/ijms19061567

**Published:** 2018-05-24

**Authors:** Claire B. Cummins, Xiaofu Wang, Omar Nunez Lopez, Gabriel Graham, Hong-Yan Tie, Jia Zhou, Ravi S. Radhakrishnan

**Affiliations:** 1 Department of Surgery, University of Texas Medical Branch, Galveston, TX 77555, USA; cbcummin@utmb.edu (C.B.C.); xfwang@utmb.edu (X.W.); oanunezl@utmb.edu (O.N.L.); 2 School of Medicine, Alabama College of Osteopathic Medicine, Dothan, AL 36303, USA; grahamgc@acom.edu; 3 Department of Anatomy, College of Basic Medicine, Zhengzhou University, Zhengzhou 450066, China; hnmzthy@163.com; 4 Department of Pharmacology and Toxicology, University of Texas Medical Branch, Galveston, TX 77555, USA; jizhou@utmb.edu

**Keywords:** hepatic fibrosis, hepatic stellate cells, luteolin, STAT3

## Abstract

Hepatic stellate cell (HSC) activation is responsible for hepatic fibrogenesis and is associated with an overexpression of transcription 3 (STAT3). Luteolin, a common dietary flavonoid with potent anti-inflammatory properties, has previously demonstrated antifibrogenic properties in HSCs but the mechanism has not been fully elucidated. Activated human and rat hepatic stellate cell lines LX-2 and HSC-T6 were used to study the effects of luteolin on HSCs. Cellular proteins were determined by western blot and immunofluorescence. Cell proliferation was assessed with Alamar Blue assay. Luteolin significantly decreased LX-2 and HSC-T6 cell viability in a time-and-dose-dependent manner, as well as decreased HSC end-products α-smooth muscle actin (α-SMA), collagen I, and fibronectin. Luteolin decreased levels of total and phosphorylated STAT3, suppressed STAT3 nuclear translocation and transcriptional activity, and attenuated expression of STAT3-regulated proteins c-myc and cyclin D1. STAT3 specific inhibitors stattic and SH-4-54 demonstrated similar effects on HSC viability and α-SMA production. In LX-2 and HSC-T6 cells, luteolin demonstrates a potent ability to inhibit hepatic fibrogenesis via suppression of the STAT3 pathway. These results further elucidate the mechanism of luteolin as well as the effect of the STAT3 pathway on HSC activation.

## 1. Introduction

Hepatic fibrosis is a wound-healing response that is the result of hepatic stellate cell (HSC) activation and subsequent excess extracellular matrix (ECM) deposition. The ECM is a hydrated gel with several components, including up to 30% collagens, as well as elastins, fibronectins, laminins, and proteoglycans [1]. In the quiescent state, HSCs participate in ECM homeostasis, vasoregulation, and metabolic homeostasis [2]. HSC activation leads to cell proliferation and over-expression of α-smooth muscle actin (α-SMA), collagens I and III, and over-expression of multiple cytokines [3,4,5,6,7]. HSCs are activated by a variety of stimuli, including transforming growth factor ß1 (TGF-ß), nuclear factor kappa light-chain enhancer of activated B cells (NF-κB), lipopolysaccharide (LPS), and tissue hypoxia [8].

The signal transducer and activator of transcription 3 (STAT3) is a transcription factor that is responsible for regulation of cell growth and survival. STAT3 is associated with liver injury, inflammation, and regeneration [9,10]. Activation of STAT3 in HSCs can help promote HSC survival, proliferation, and activation, thus promoting the formation of hepatic fibrosis [11,12,13]. Multiple studies have demonstrated that apoptosis of activated HSCs results in accelerated recovery from liver fibrosis [14] and work from our lab has confirmed that inhibition of STAT3 results in HSC apoptosis [15].

Luteolin is one of the most common flavonoids present in edible plants that have been used in traditional medicine to treat a wide variety of pathologies [16]. It is present in many vegetables, fruits, and medicinal herbs, including celery, pepper, cucumber, artichoke, capers, pomegranate, peppermint, thyme, oregano, and rooibos teas [16]. Typical daily intake of luteolin in the US is 20–22 mg and a Chinese population demonstrated plasma concentrations of 101 ± 99 nmol/L [17,18]. Luteolin has been shown to have antifibrogenic effects in activated HSCs, though the mechanism has not been fully elucidated [16,19]. Luteolin is also known to have strong antioxidant, radical scavenging, and anti-inflammatory properties [16]. Pilot clinical trials have been conducted both with natural medicines containing luteolin and concentrated luteolin extract and no significant adverse effects have been noted [20,21]. Luteolin has been shown to alleviate alcohol-induced hepatic steatosis in mice and to inhibit cytochrome P450 (CYP) enzymes [22,23]. Additionally, multiple studies have demonstrated that luteolin inhibits the STAT3 pathway in a variety of cell types, including pancreatic cancer cells, lung adenocarcinoma cells, gastric cancer cells, and breast cancer cells [24,25,26,27,28,29].

Taking these lines of evidence together, we hypothesized that luteolin would inhibit hepatic fibrogenesis via STAT3 inhibition.

## 2. Results

### 2.1. Lutelin Inhibits LX-2 and HSC-T6 Cell Proliferation

**Figure 1:**
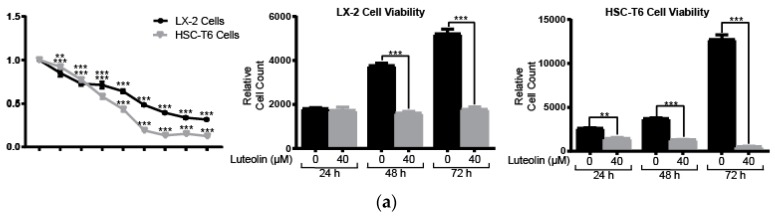

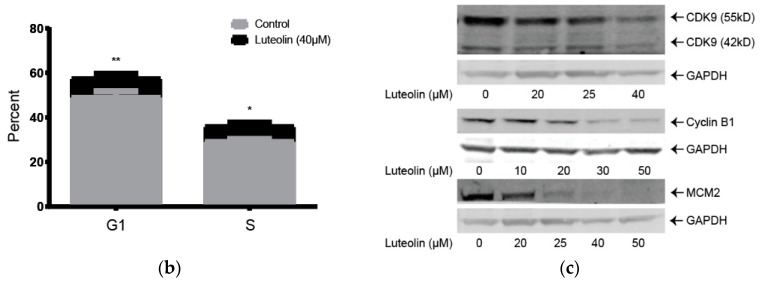
Luteolin inhibits LX-2 and HSC-T6 cell proliferation. (**a**) LX-2 and HSC-6 cell viability after treatment with a series of concentrations of luteolin for 48 h (left panel). LX-2 (middle panel) and HSC-T6 (right panel) cell viability after treatment with vehicle or 40 µM of luteolin for 24, 48, or 72 h; (**b**) Percent of cells in G1 and S phase after treatment with vehicle or 40 µM of luteolin for 24 h; (**c**) Western blots with LX-2 whole cell lysate after incubation with vehicle or 40 µM of luteolin for 24 h for cell cycle regulatory proteins cyclin-dependent kinase 9 (CDK9), cyclin B1 and minichromosome maintenance protein 2 (MCM2). *p*-Values shown compared to vehicle. The results are representative of at least 3 independent experiments. All summary bar and line graphs are presented as mean ± SEM, with significance denoted as follows *: *p* < 0.05, **: *p* < 0.01, ***: *p* < 0.001.

We assessed HSC viability after luteolin administration using Alamar Blue assay. Luteolin inhibits LX-2 cell viability in a dose-dependent manner with a half-maximal inhibitory concentration (IC_50_) of 20.22 µM. LX-2 cell viability was significantly decreased at concentrations greater than 5 µM (*p* < 0.001) (Figure 1a). Luteolin inhibits HSC-T6 cell viability in a dose-dependent manner with an IC_50_ of 15.95 µM. HSC-T6 cell viability was significantly decreased at concentrations greater than 5 µM (*p* < 0.01) (Figure 1a). Luteolin also significantly inhibited LX-2 and HSC-T6 cell viability in a time-dependent manner. Treatment with Luteolin significantly inhibited LX-2 cell viability at 48 and 72 h (*p* < 0.001). HSC-T6 cell viability was significantly inhibited by luteolin at 24, 48, and 72 h (*p* < 0.001) (Figure 1a).

We also examined the effects of luteolin on the cell cycle and found that luteolin induces cell cycle arrest in LX-2 cells. As indicated in Figure 1b, Luteolin significantly increased the number of cells in the G1 and S phases compared to control (*p* < 0.01 and *p* < 0.05, respectively). Luteolin also down-regulates cell cycle regulation proteins in a dose-dependent manner. Cell cycle regulators cyclin-dependent kinase 9 (CDK9) and cyclin B1, as well as DNA replication licensing factor minichromosome maintenance protein 2 (MCM2), are all noticeably reduced in a dose-dependent manner in LX-2 cells after administration of luteolin (Figure 1c). STAT3-regulated cell cycle proteins c-myc and cyclin D1 were also markedly decreased, as presented in a future figure.

### 2.2. Luteolin Induces HSC Apoptosis and Attenuates α-SMA Expression

**Figure 2:**
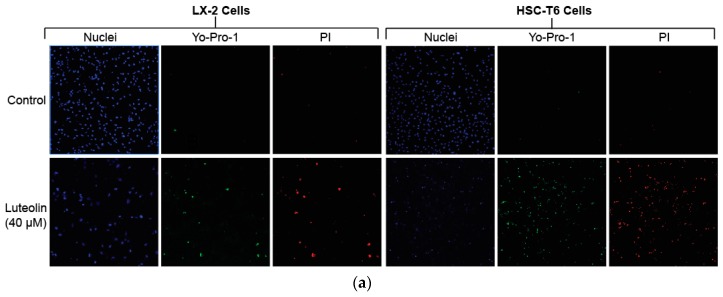

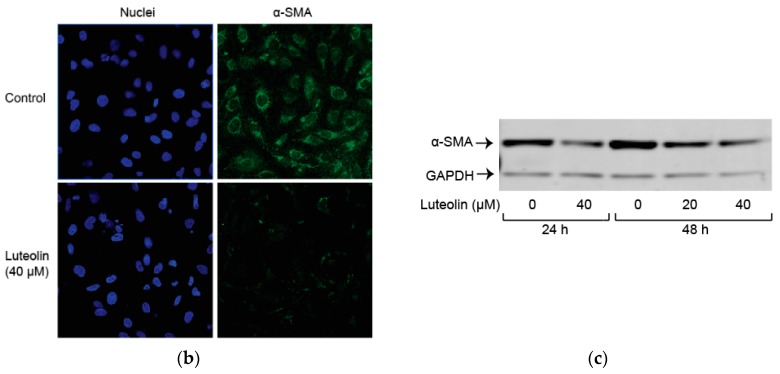
Luteolin induces hepatic stellate cell (HSC) apoptosis and attenuates α-smooth muscle actin (α-SMA) expression. (**a**) Fluorescence staining for Yo-Pro-1 and PI after treatment with vehicle or 40 µM of luteolin for 24 h in both LX-2 and HSC-T6 cells; (**b**) Immunofluorescence staining for α-SMA after treatment with vehicle or 40 µM of luteolin for 24 h in LX-2 cells; (**c**) Western blot with LX-2 whole cell lysate after incubation for 24 or 48 h with vehicle or varying concentrations of luteolin for α-SMA. The results are representative of at least 3 independent experiments.

To detect apoptosis in LX-2 and HSC-T6 cells, we used fluorescence staining for early apoptotic marker Yo-Pro-1 and late apoptotic marker propidium iodide (PI). Treatment with 40 µM of luteolin demonstrated markedly increased early and late apoptosis in both LX-2 and HSC-T6 cell lines (Figure 2a). We also studied α-smooth muscle actin (α-SMA) levels, which is a surrogate marker for HSC activation. Immunofluorescence staining for α-SMA in LX-2 cells demonstrated a marked attenuation of α-SMA levels after treatment with 40 µM of luteolin (Figure 2b). This result was confirmed with western blot, which demonstrated a time-and-dose-dependent attenuation of α-SMA expression in LX-2 cells after treatment with luteolin at varying concentrations and time points (Figure 2c).

### 2.3. Luteolin Suppresses the STAT3 Pathway

**Figure 3:**
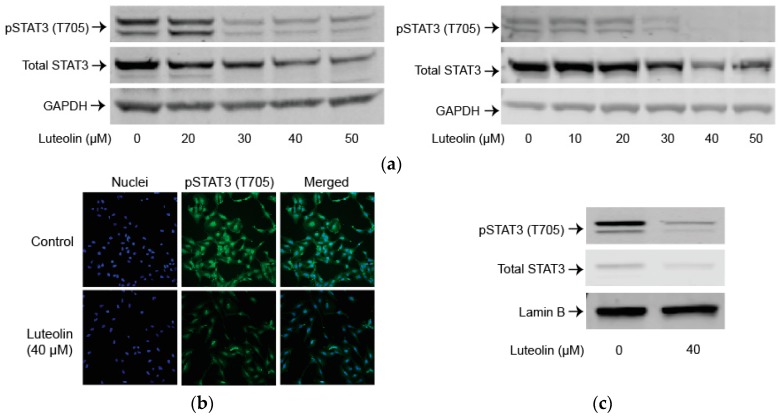

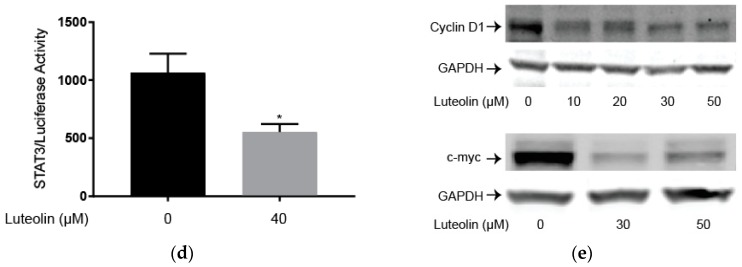
Luteolin suppresses the transcription 3 (STAT3) pathway. (**a**) Western blot with LX-2 (left panel) or HSC-T6 (right panel) whole cell lysate after incubation with varying concentrations of luteolin for 24 h for phosphorylated STAT3 (Tyr705) or total STAT3; (**b**) Immunofluorescence staining for phosphorylated STAT3 (Tyr705) after treatment with vehicle or 40 µM of luteolin for 24 h in LX-2 cells; (**c**) Western blot with LX-2 nuclear fraction after incubation with vehicle or 40 µM of luteolin for 24 h for phosphorylated STAT3 (Tyr705) or total STAT3; (**d**) Luciferase assay for LX-2 cells transfected with STAT3/Cignal Reporter to measure STAT3 transcriptional activity; (**e**) Western blots with LX-2 whole cell lysate after incubation with varying concentrations of luteolin for 24 h for STAT3 regulated proteins cyclin D1 and c-myc. *p*-Values shown compared to vehicle. The results are representative of at least 3 independent experiments. All summary bar and line graphs are presented as mean ± SEM, with significance denoted as follow *: *p* < 0.05.

To examine the role of the STAT3 pathway after luteolin administration, we looked at total and phosphorylated levels of STAT3. Luteolin markedly decreased phosphorylated STAT3 (Tyr705) levels in a dose-dependent manner. Luteolin also markedly decreased total STAT3 in a dose-dependent manner (Figure 3a). Immunofluorescence for phosphorylated STAT3 (Tyr705) confirmed that levels of STAT3 were noticeably reduced after treatment with 40 µM of luteolin (Figure 3b). Nuclear phosphorylated STAT3 levels significantly decreased after administration of 40 µM of luteolin (*p* < 0.05) (Figure 3c).

We performed luciferase assay to examine STAT3 transcriptional activity. Significantly lower STAT3 transcriptional activity was demonstrated after treatment with 40 µM of luteolin (*p* < 0.05) (Figure 3d).

Finally, we examined STAT3 activation-regulated proteins cyclin D1 and c-myc. Luteolin markedly decreased cyclin D1 and c-myc in a dose-dependent manner (Figure 3e).

### 2.4. STAT3 Specific Inhibitors Suppress HSC Activation

**Figure 4:**
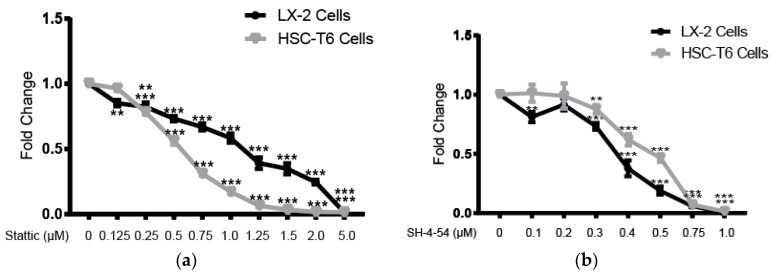

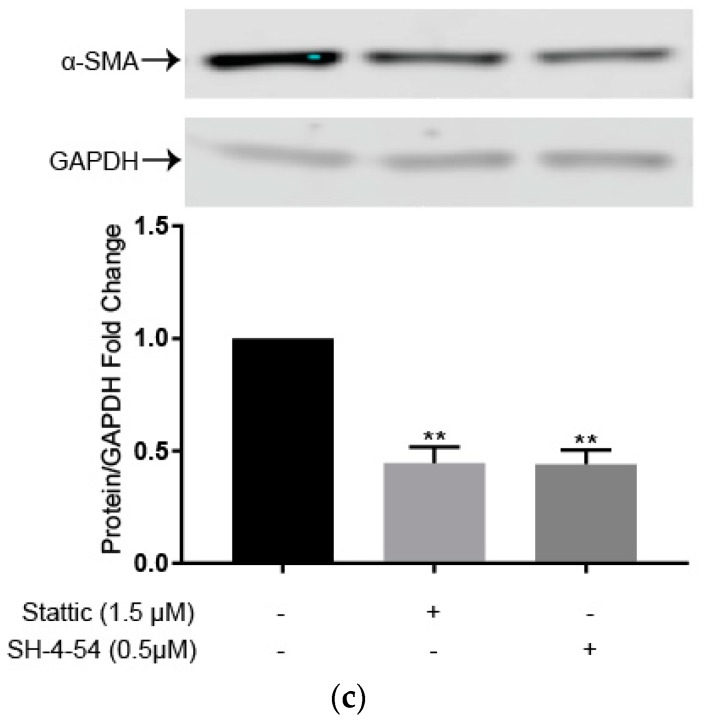
STAT3 specific inhibitors suppress HSC activation. (**a**) LX-2 and HSC-T6 cell viability after treatment with varying concentrations of STAT3 specific inhibitor stattic for 48 h; (**b**) LX-2 and HSC-T6 cell viability after treatment with varying concentrations of STAT3 specific inhibitor SH-4-54 for 48 h; (**c**) Western blot with LX-2 whole cell lysate after incubation with vehicle, 1.5 µM of stattic, or 0.5 µM of SH-4-54 for 72 h for α-SMA. Densitometric analyses of bands were quantified and data expressed as fold of control normalized to Glyceraldehyde 3-phosphate dehydrogenase (GAPDH). *P*-values shown compared to vehicle. The results are representative of at least 3 independent experiments. All summary bar and line graphs are presented as mean ± SEM, with significance denoted as follows **: *p* < 0.01, ***: *p* < 0.001.

We used STAT3 specific inhibitors stattic and SH-4-54 to confirm that inhibition of STAT3 leads to decreased cell viability, similar to luteolin administration. Stattic inhibited LX-2 cell viability in a dose-dependent manner with an IC_50_ of 1.38 µM. LX-2 cell viability was significantly decreased at stattic concentrations greater than 0.125 µM (*p* < 0.01). Stattic inhibited HSC-T6 cell viability in a dose-dependent manner with an IC_50_ of 0.54 µM. HSC-T6 cell viability was significantly decreased at stattic concentrations greater than 0.25 µM (*p* < 0.001) (Figure 4a). A second STAT3 specific inhibitor, SH-4-54, inhibited LX-2 cell viability in a dose-dependent manner with an IC_50_ of 0.37 µM. LX-2 cell viability was significantly decreased at SH-4-54 concentrations greater than 0.3 µM (*p* < 0.001). SH-4-54 inhibited HSC-T6 cell viability in a dose-dependent manner with an IC_50_ of 0.46 µM. HSC-T6 cell viability was significantly decreased at SH-4-54 concentrations greater than 0.3 µM (*p* < 0.01) (Figure 4b).

STAT3 specific inhibitors stattic and SH-4-54 also significantly attenuated a surrogate marker of activation α-SMA, similar to luteolin. Treatment with 1.5 µM of stattic or treatment with 0.5 µM of SH-4-54 resulted in significantly reduced levels of α-SMA (*p* < 0.01 for both) (Figure 4c). 

### 2.5. Luteolin Inhibits Endogenous and TGF-ß Induced ECM Proteins

**Figure 5:**
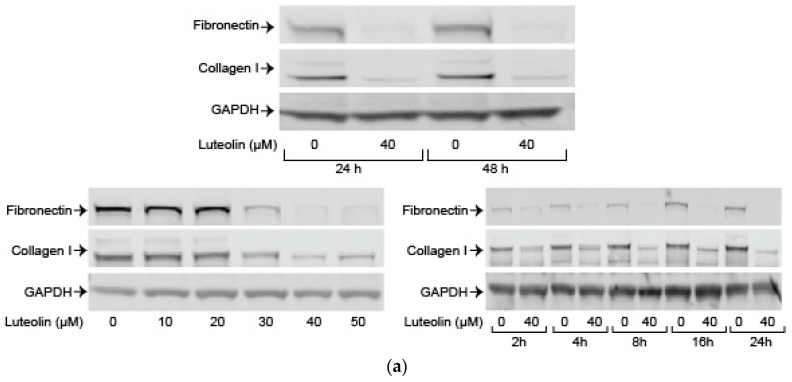

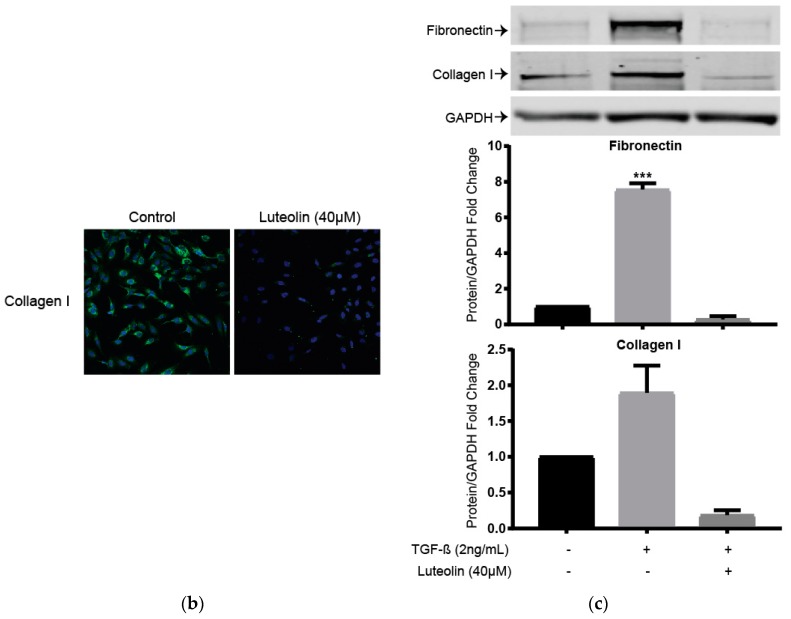
Luteolin inhibits endogenous and transforming growth factor ß1(TGF-ß) induced excess extracellular matrix (ECM) proteins. (**a**) Western blot with LX-2 (top panel) whole cell lysate after incubation with vehicle or 40 µM of luteolin for fibronectin and collagen I. HSC-T6 (bottom panels) whole cell lysate after incubation for the indicated number of hours with varying concentrations of luteolin for fibronectin and collagen I; (**b**) Immunofluorescence staining for collagen I after treatment with vehicle or 40 µM of luteolin for 24 h in LX-2 cells; (**c**) Western blot with LX-2 whole cell lysate after incubation with vehicle, 2 ng/mL of TGF-ß, or both 2 ng/mL of TGF-ß and 40 µM of luteolin for fibronectin and collagen I. Densitometric analyses of bands were quantified and data expressed as fold of control normalized to GAPDH. *P*-values shown compared to vehicle. The results are representative of at least 3 independent experiments. All summary bar and line graphs are presented as mean ± SEM, with significance denoted as follow ***: *p* < 0.001.

Finally, we examined the effects of luteolin on endogenous and TGF-ß induced levels of key ECM components collagen I and fibronectin. Luteolin attenuated endogenous collagen I and fibronectin in a dose-and-time-dependent fashion (Figure 5a). Immunofluorescence staining for collagen I demonstrated marked attenuation after administration of 40 µM of luteolin (Figure 5b). We examined the effects of luteolin after administration of TGF-ß, one of the most potent and well-described activators of HSCs. Luteolin administration significantly decreased TGF-ß induced levels of fibronectin (*p* < 0.01) and markedly reduced TGF-ß induced levels of collagen I (Figure 5c).

## 3. Discussion

Our results demonstrate that luteolin is an effective potential antifibrogenic agent in LX-2 and HSC-T6 cells and its effects are mediated via suppression of the STAT3 pathway. Luteolin significantly decreases cell viability and induces apoptosis and cell cycle arrest in these cell lines. Luteolin also decreases ECM components collagen I, α-SMA, and fibronectin. In addition, we demonstrated that the STAT3 pathway is impaired by luteolin, suggesting a possible mechanism for its antifibrogenic properties. Use of a selective STAT3 inhibitor confirms that STAT3 pathway inhibition results in the same antifibrogenic effects in HSC as those seen with luteolin.

Luteolin significantly decreased HSC cell viability in a dose-and-time dependent manner. In addition, Yo-Pro-1 and PI staining demonstrated a marked increase in early and late apoptosis in luteolin-treated HSCs. Multiple studies have demonstrated that apoptosis of HSCs has resulted in accelerated recovery from liver fibrosis [14]. Hepatic fibrosis is a slow, chronic process that typically evolves over decades and can ultimately lead to hepatic cirrhosis [30]. While traditional treatments have focused on reducing exposure to the etiological agents, more recent research has been focused on identifying agents which can prevent progression or induce regression of hepatic fibrosis [31]. Luteolin’s potent antifibrogenic properties indicate that it could be an effective potential antifibrogenic agent after further study.

Luteolin also induces cell cycle arrest in HSCs. Administration of luteolin significantly increases the percentage of cells in G1 or S phase, as well as downregulates cell cycle regulatory proteins CDK9, cyclin B1, MCM2, cyclin D1, and c-myc. The mechanism of this cell cycle arrest could be related to STAT3 pathway inhibition. When STAT3 is phosphorylated, it forms dimers and moves from the cytoplasm to the nucleus and stimulates transcription of STAT3 target genes, including cyclin D1 and c-myc [32]. The cell cycle has 5 distinct phases: G0 (quiescence), G1 (growth), S (synthesis), G2 (gap), and M (mitosis). The G1 checkpoint is vital to ensure that everything is ready for DNA synthesis and once it is crossed, the cell cycle will continue until completed. This checkpoint is regulated by the coordinated action of cell cycle regulatory proteins (CDKs) in association with their specific regulatory cyclin proteins [33]. Synthesis of cyclin D1 is stimulated in the early G1 phase and activates and binds G1 specific CDK proteins [33]. Downregulation of cyclin D1 is associated with G1 phase arrest [33]. Similarly, downregulation of c-myc, a regulator gene which codes for a DNA transcription factor via suppression of the STAT3 pathway, could be contributing both to the luteolin-induced apoptosis seen in HSCs as well as the cell cycle arrest. 

Treatment with luteolin results in a dose-and-time dependent attenuation of α-SMA, collagen I, and fibronectin, which are all key ECM proteins expressed by activated HSCs [3,4,5,6,7]. α-SMA in particular is a surrogate marker of HSC activation. Attenuation of these key proteins confirms the antifibrogenic properties of luteolin in an in vitro model. Our results also demonstrate that luteolin inhibits both endogenous and TGF-ß induced expression of ECM proteins. It is well known that TGF-ß is the most potent fibrogenic factor responsible for HSC activation. Luteolin inhibits both endogenous and TGF-ß induced expression of ECM proteins, providing stronger evidence of the antifibrogenic properties of luteolin.

Our results demonstrate that STAT3 inhibition in LX-2 and HSC-T6 cells results in antifibrogenic effects. This is consistent with other studies that have demonstrated that the STAT3 pathway is an important player in HSC activation and that STAT3 represents a potential therapeutic target for antifibrogenic therapy [13,34,35]. We demonstrated that luteolin resulted in downregulation of the STAT3 pathway. In addition to the originally described activation by phosphorylation of the tyrosine 705 residue of STAT3, it has also been demonstrated that dimers of non-phosphorylated STAT3 can exist and be active and phosphorylation of STAT3 (Tyr705), STAT3 (Ser727), or both can produce transcriptional activity [36,37,38]. We have demonstrated that luteolin inhibits STAT3 phosphorylation, prevents STAT3 nuclear translocation, decreases STAT3 transcriptional activity, and downregulates STAT3-regulated genes. This evidence chain supports the conclusion that activation of the STAT3 pathway promotes fibrogenic behavior in HSCs.

Our results also demonstrate that the administration of STAT3 specific inhibitors, stattic and SH-4-54, significantly decrease HSC viability and significantly attenuate expression of α-SMA. Stattic is a nonpeptidic small molecule that has been shown to selectively inhibit the function of the STAT3 SH2 domain, thereby selectively inhibiting activation, dimerization and nuclear translocation of STAT3 [39]. SH-4-54 is a small molecule which also binds to the SH2 domain of STAT3, and inhibits STAT3 phosphorylation [40]. These results further confirm that the inhibition of the STAT3 pathway has a potent antifibrogenic effect.

The overexpression of STAT3 in hepatic fibrosis may have model-dependent and cell-specific functions [41,42]. Murine models involving carbon tetrachloride induced fibrosis have demonstrated that STAT3 activation prevents hepatic fibrosis, while activation of STAT3 in the dimethylnitrosamine induced fibrosis model promotes fibrosis [42,43,44,45]. It is likely that the role of STAT3 in HSCs depends on the inflammatory context. IL-22, an anti-inflammatory cytokine, induces cellular senescence in HSCs via activation of the STAT3 pathway [46]. However, pro-inflammatory cytokines, such as IL-6 and leptin activate STAT3 in HSCs and can result in fibrogenic behavior [9,13,34,35]. Hepatic injury, by a variety of etiologies, is the typical inciting factor for the development of hepatic fibrosis and cirrhosis. After injury, HSCs are exposed to a pro-inflammatory microenvironment [47,48,49]. This indicates that inhibition of the STAT3 pathway under these pro-inflammatory conditions will combat hepatic fibrosis, which is consistent with our results.

## 4. Materials and Methods

### 4.1. Reagents

Cell culture mediums and trypsin were purchased from Life Technology Corp. (Carlsbad, CA, USA). Luteolin was purchased from Sigma-Aldrich (Cat#L9283St. Louis, MO, USA). TGF-ß1 was purchased from R&D Systems (Cat#240-B, Minneapolis, MN, USA). Stattic was purchased from Sigma-Aldrich (Cat#S7947). SH-4-54 was purchased from Millipore Sigma (Cat#509105, Burlington, MA, USA). 

### 4.2. Cell Culture

The human immortalized HSC line LX-2 and rat immortalized HSC line HSC-T6 were a gift from Dr. Scott Friedman (Mount Sinai Medical Center, NY, USA) and cultured at 37 °C with 5% CO_2_ in Dulbecco’s modified Eagle’s medium (DMEM) with a high glucose concentration (4.5 g/L) supplemented with 5% fetal bovine serum (FBS) and 1% penicillin/streptomycin. All experiments were performed on cells within 6 weeks of culture from liquid nitrogen. Human and rat cell lines were used to ensure that these effects were not limited to a single cell line.

### 4.3. Cell Viability Assay

Cell viability was assessed using Alamar Blue Cell Viability Reagent (Cat#DAL1025) purchased from Life Technologies (Grand Island, NY, USA) and by following the manufacturer’s instructions. Fluorescence intensity was monitored using a SpectraMax M5 microplate reader from Molecular Devices, LLC (Sunnyvale, CA, USA) with excitation and emission wavelengths set at 544 and 590 nm, respectively. Assay was performed in triplicate and repeated at least 3 times.

### 4.4. Detection of Apoptosis

For the detection of apoptosis by Yo-Pro-1 (Cat#Y3603, Molecular Probes, Eugene, OR, USA) and propidium iodide (PI) (Cat#P3566, Life Technologies Corporation), cells were seeded in 24-well plates with 0.25 × 10^5^ cells/well. The next day, cells were treated with 40 µM of luteolin for 24 h. After being washed with phosphate buffered saline (PBS), cells were incubated with 1 µM of Yo-Pro-1 or PI for 1 h. Yo-Pro-1 and PI uptake were determined by confocal microscope (Nikon Instruments Inc., Melville, NY, USA)

### 4.5. Immunofluorescence Staining

Immunofluorescence staining was performed as previously described [15] with α-SMA (Cat#A5228, Sigma-Aldrich), phosphorylated STAT3 (Tyr705) (Cat#9145, Cell Signaling, Danvers, MA, USA) antibodies. After the indicated treatments and staining, the cells were visualized by Nikon Eclipse Ti confocal microscope at 20× magnification (Nikon Instruments Inc.).

### 4.6. Luciferase Assay

STAT3 transcriptional activity was determined by luciferase assay using STAT3/Cignal Reporter assay kit (Cat#CCS-9028L) purchased from Qiagen (Hilden, Germany) by following the manufacturer’s instructions. Assay was repeated three times.

### 4.7. Western Immunoblotting

Whole cell extracts were prepared using radioimmunoprecipitation assay buffer (RIPA buffer) (Thermo Fischer Scientific, Inc., Waltham, MA, USA) with 1% Halt protease inhibitor cocktail and 1% Halt phosphatase inhibitor cocktails (Thermo Fischer Scientific, Inc.). Nuclear protein was isolated as previously described [50]. The protein concentration was measured and quantified by the Bradford method. 10–30 g of protein were fractionated by sodium dodecyl sulfate-polyacrylamide gel electrophoresis (SDS-PAGE) (Life technologies Corporation) under denaturing conditions and then electro-transferred to a polyvinylidene fluoride (PVDF) membrane. After being blocked with blocking buffer (LI-COR, Inc., Lincoln, NE, USA) the membrane was probed with the indicated primary antibody diluted with blocking buffer. Membranes were washed 3 times with phosphate buffered saline with 0.1% Tween 20 (PBST), and incubated 1 h with infrared fluorescent dye (IRDye) 680-conjugated anti-mouse or IRDye 800-conjugated anti-rabbit Ab (LI-COR, Inc.). Finally, the membranes were washed three times with PBST, and signals were scanned and visualized by Odyssey Infrared Imaging System (LI-COR, Inc.). Antibodies used included cyclin dependent kinase 9 (CDK9) (Cat#2316), cyclin B1 (Cat#4138), phosphorylated STAT3 Tyr705 (Cat#9145), and STAT3 (Cat#4904) purchased from Cell Signaling. Antibodies for Minichromosome maintenance protein 2 (MCM2) (Cat#9839) and fibronectin (Cat#6952) were purchased from Santa Cruz Inc. (Santa Cruz, CA, USA). Antibodies for cyclin D1 (Cat#2261-1) and c-myc (Cat#1472-1) were purchased from Epitomics (Burlingame, CA, USA). α-SMA (Cat#A5228) antibodies were purchased from Sigma-Aldrich (St. Louis, MO, USA) and collagen I (Cat #600-401-103) antibodies were purchased from Rockland (Pottstown, PA, USA). All blots were repeated at least 3 times. 

### 4.8. Statistical Analysis

Statistical analysis was performed using GraphPad Prism 7.0 from GraphPad Software Inc. (La Jolla, CA, USA). Where indicated, one-way ANOVA with Sidak’s multiple comparisons test or *t*-test were used. All summary bar and line graphs are presented as mean ± SEM, with significance denoted as follows *: *p* < 0.05, **: *p* < 0.01, ***: *p* < 0.001.

## 5. Conclusions

In activated human and rat HSCs, luteolin has demonstrated a significant ability to potentially inhibit hepatic fibrosis. These effects are mediated by suppression of the STAT3 pathway, further elucidating the mechanism of luteolin, as well the effect of the STAT3 pathway on HSC activation.

## Abbreviations

HSC	Hepatic stellate cell
ECM	Extracellular matrix
TGF-ß	Transforming growth factor ß1
STAT3	Signal transducer and activator of transcription 3
IC_50_	Half maximal inhibitory concentration
CDK9	Cyclin dependent kinase 9
MCM2	Minichromosome maintenance protein 2
PI	Propidium iodide

## References

[1] Roderfeld M. (2017). Matrix metalloproteinase functions in hepatic injury and fibrosis. Matrix Biol..

[2] Alter G., Heckerman D., Schneidewind A., Fadda L., Kadie C.M., Carlson J.M., Oniangue-Ndza C., Martin M., Li B., Khakoo S.I. (2011). HIV-1 adaptation to NK-cell-mediated immune pressure. Nature.

[3] Lemoinne S., Cadoret A., El Mourabit H., Thabut D., Housset C. (2013). Origins and functions of liver myofibroblasts. Biochim. Biophys. Acta.

[4] Liu Y., Wang Z., Kwong S.Q., Lui E.L.H., Friedman S.L., Li F.R., Lam R.W.C., Zhang G.C., Zhang H., Ye T. (2011). Inhibition of PDGF, TGF-beta, and Abl signaling and reduction of liver fibrosis by the small molecule Bcr-Abl tyrosine kinase antagonist Nilotinib. J. Hepatol..

[5] Lee S.H., Seo G.S., Park Y.N., Yoo T.M., Sohn D.H. (2004). Effects and regulation of osteopontin in rat hepatic stellate cells. Biochem. Pharmacol..

[6] Ramm G.A., Shepherd R.W., Hoskins A.C., Greco S.A., Ney A.D., Pereira T.N., Bridle K.R., Doecke J.D., Meikle P.J., Turlin B. (2009). Fibrogenesis in pediatric cholestatic liver disease: Role of taurocholate and hepatocyte-derived monocyte chemotaxis protein-1 in hepatic stellate cell recruitment. Hepatology.

[7] Fang S., Yuan J., Shi Q., Xu T., Fu Y., Wu Z., Guo W. (2017). Downregulation of UBC9 promotes apoptosis of activated human LX-2 hepatic stellate cells by suppressing the canonical NF-kappaB signaling pathway. PLoS ONE.

[8] Tsukamoto H., Zhu N.L., Wang J., Asahina K., Machida K. (2012). Morphogens and hepatic stellate cell fate regulation in chronic liver disease. J. Gastroenterol. Hepatol..

[9] Kong X., Horiguchi N., Mori M., Gao B. (2012). Cytokines and STATs in Liver Fibrosis. Front. Physiol..

[10] Wang H., Lafdil F., Kong X., Gao B. (2011). Signal transducer and activator of transcription 3 in liver diseases: A novel therapeutic target. Int. J. Biol. Sci..

[11] Jiang J.X., Mikami K., Venugopal S., Li Y., Torok N.J. (2009). Apoptotic body engulfment by hepatic stellate cells promotes their survival by the JAK/STAT and Akt/NF-kappaB-dependent pathways. J. Hepatol..

[12] Parola M., Marra F. (2011). Adipokines and redox signaling: Impact on fatty liver disease. Antioxid. Redox Signal..

[13] Su T.H., Shiau C.W., Jao P., Liu C.H., Liu C.J., Tai W.T., Jeng Y.M., Yang H.C., Tseng T.C., Huang H.P. (2015). Sorafenib and its derivative SC-1 exhibit antifibrotic effects through signal transducer and activator of transcription 3 inhibition. Proc. Natl. Acad. Sci. USA.

[14] Elsharkawy A.M., Oakley F., Mann D.A. (2005). The role and regulation of hepatic stellate cell apoptosis in reversal of liver fibrosis. Apoptosis.

[15] Nunez Lopez O., Bohanon F.J., Wang X., Ye N., Corsello T., Rojas-Khalil Y., Chen H., Chen H., Zhou J., Radhakrishnan R.S. (2016). STAT3 Inhibition Suppresses Hepatic Stellate Cell Fibrogenesis: HJC0123, a Potential Therapeutic Agent for Liver Fibrosis. RSC Adv..

[16] Lopez-Lazaro M. (2009). Distribution and biological activities of the flavonoid luteolin. Mini Rev. Med. Chem..

[17] Chun O.K., Chung S.J., Song W.O. (2007). Estimated dietary flavonoid intake and major food sources of U.S. adults. J. Nutr..

[18] Zhang Y., Cao J., Chen W., Yang J., Hao D., Zhang Y., Chang P., Zhao X. (2010). Reproducibility and relative validity of a food frequency questionnaire to assess intake of dietary flavonol and flavone in Chinese university campus population. Nutr. Res..

[19] Li J., Li X., Xu W., Wang S., Hu Z., Zhang Q., Deng X., Wang J., Zhang J., Guo C. (2015). Antifibrotic effects of luteolin on hepatic stellate cells and liver fibrosis by targeting AKT/mTOR/p70S6K and TGFbeta/Smad signalling pathways. Liver Int..

[20] Perry N.S., Bollen C., Perry E.K., Ballard C. (2003). Salvia for dementia therapy: review of pharmacological activity and pilot tolerability clinical trial. Pharmacol. Biochem. Behav..

[21] Taliou A., Zintzaras E., Lykouras L., Francis K. (2013). An open-label pilot study of a formulation containing the anti-inflammatory flavonoid luteolin and its effects on behavior in children with autism spectrum disorders. Clin. Ther..

[22] Cao L., Kwara A., Greenblatt D.J. (2017). Metabolic interactions between acetaminophen (paracetamol) and two flavonoids, luteolin and quercetin, through in-vitro inhibition studies. J. Pharm. Pharmacol..

[23] Liu G., Zhang Y., Liu C., Xu D., Zhang R., Cheng Y., Pan Y., Huang C., Chen Y. (2014). Luteolin alleviates alcoholic liver disease induced by chronic and binge ethanol feeding in mice. J. Nutr..

[24] Aneknan P., Kukongviriyapan V., Prawan A., Kongpetch S., Sripa B., Senggunprai L. (2014). Luteolin arrests cell cycling, induces apoptosis and inhibits the JAK/STAT3 pathway in human cholangiocarcinoma cells. Asian Pac. J. Cancer Prev..

[25] Fu J., Chen D., Zhao B., Zhao Z., Zhou J., Xu Y., Xin Y., Liu C., Luo L., Yin Z. (2012). Luteolin induces carcinoma cell apoptosis through binding Hsp90 to suppress constitutive activation of STAT3. PLoS ONE.

[26] Huang X., Dai S., Dai J., Xiao Y., Bai Y., Chen B., Zhou M. (2015). Luteolin decreases invasiveness, deactivates STAT3 signaling, and reverses interleukin-6 induced epithelial-mesenchymal transition and matrix metalloproteinase secretion of pancreatic cancer cells. OncoTargets Ther..

[27] Sonoki H., Tanimae A., Endo S., Matsunaga T., Furuta T., Ichihara K., Ikari A. (2017). Kaempherol and Luteolin Decrease Claudin-2 Expression Mediated by Inhibition of STAT3 in Lung Adenocarcinoma A549 Cells. Nutrients.

[28] Song S., Su Z., Xu H., Niu M., Chen X., Min H., Zhang B., Sun G., Xie S., Wang H. (2017). Luteolin selectively kills STAT3 highly activated gastric cancer cells through enhancing the binding of STAT3 to SHP-1. Cell Death Dis..

[29] Yang M.Y., Wang C.J., Chen N.F., Ho W.H., Lu F.J., Tseng T.H. (2014). Luteolin enhances paclitaxel-induced apoptosis in human breast cancer MDA-MB-231 cells by blocking STAT3. Chem. Biol. Interact..

[30] Lee U.E., Friedman S.L. (2011). Mechanisms of hepatic fibrogenesis. Best Pract. Res. Clin. Gastroenterol..

[31] Schuppan D., Kim Y.O. (2013). Evolving therapies for liver fibrosis. J. Clin. Investig..

[32] Wang S.W., Sun Y.M. (2014). The IL-6/JAK/STAT3 pathway: Potential therapeutic strategies in treating colorectal cancer (Review). Int. J. Oncol..

[33] Li A., Wang J., Wu M., Zhang X., Zhang H. (2015). The inhibition of activated hepatic stellate cells proliferation by arctigenin through G0/G1 phase cell cycle arrest: Persistent p27(Kip1) induction by interfering with PI3K/Akt/FOXO3a signaling pathway. Eur. J. Pharmacol..

[34] Xiang D.M., Sun W., Ning B.F., Zhou T.F., Li X.F., Zhong W., Cheng Z., Xia M.Y., Wang X., Deng X. (2017). The HLF/IL-6/STAT3 feedforward circuit drives hepatic stellate cell activation to promote liver fibrosis. Gut.

[35] Kagan P., Sultan M., Tachlytski I., Safran M., Ben-Ari Z. (2017). Both MAPK and STAT3 signal transduction pathways are necessary for IL-6-dependent hepatic stellate cells activation. PLoS ONE.

[36] Li C., Iness A., Yoon J., Grider J.R., Murthy K.S., Kellum J.M., Kuemmerle J.F. (2015). Noncanonical STAT3 activation regulates excess TGF-beta1 and collagen I expression in muscle of stricturing Crohn’s disease. J. Immunol..

[37] Huang Y., Qiu J., Dong S., Redell M.S., Poli V., Mancini M.A., Tweardy D.J. (2007). Stat3 isoforms, alpha and beta, demonstrate distinct intracellular dynamics with prolonged nuclear retention of Stat3beta mapping to its unique C-terminal end. J. Biol. Chem..

[38] Wake M.S., Watson C.J. (2015). STAT3 the oncogene—Still eluding therapy?. FEBS J..

[39] Schust J., Sperl B., Hollis A., Mayer T.U., Berg T. (2006). Stattic: A small-molecule inhibitor of STAT3 activation and dimerization. Chem. Biol..

[40] Haftchenary S., Luchman H.A., Jouk A.O., Veloso A.J., Page B.D., Cheng X.R., Dawson S.S., Grinshtein N., Shahani V.M., Kerman K. (2013). Potent Targeting of the STAT3 Protein in Brain Cancer Stem Cells: A Promising Route for Treating Glioblastoma. ACS Med. Chem. Lett..

[41] Xu M.Y., Hu J.J., Shen J., Wang M.L., Zhang Q.Q., Qu Y., Lu L.G. (2014). Stat3 signaling activation crosslinking of TGF-beta1 in hepatic stellate cell exacerbates liver injury and fibrosis. Biochim. Biophys. Acta.

[42] Ogata H., Chinen T., Yoshida T., Kinjyo I., Takaesu G., Shiraishi H., Iida M., Kobayashi T., Yoshimura A. (2006). Loss of SOCS3 in the liver promotes fibrosis by enhancing STAT3-mediated TGF-beta1 production. Oncogene.

[43] Gao B., Wang H., Lafdil F., Feng D. (2012). STAT proteins—Key regulators of anti-viral responses, inflammation, and tumorigenesis in the liver. J. Hepatol..

[44] Mair M., Zollner G., Schneller D., Musteanu M., Fickert P., Gumhold J., Schuster C., Fuchsbichler A., Bilban M., Tauber S. (2010). Signal transducer and activator of transcription 3 protects from liver injury and fibrosis in a mouse model of sclerosing cholangitis. Gastroenterology.

[45] Plum W., Tschaharganeh D.F., Kroy D.C., Corsten E., Erschfeld S., Dierssen U., Wasmuth H., Trautwein C., Streetz K.L. (2010). Lack of glycoprotein 130/signal transducer and activator of transcription 3-mediated signaling in hepatocytes enhances chronic liver injury and fibrosis progression in a model of sclerosing cholangitis. Am. J. Pathol..

[46] Kong X., Feng D., Wang H., Hong F., Bertola A., Wang F.S., Gao B. (2012). Interleukin-22 induces hepatic stellate cell senescence and restricts liver fibrosis in mice. Hepatology.

[47] Casini A., Ceni E., Salzano R., Biondi P., Parola M., Galli A., Foschi M., Caligiuri A., Pinzani M., Surrenti C. (1997). Neutrophil-derived superoxide anion induces lipid peroxidation and stimulates collagen synthesis in human hepatic stellate cells: role of nitric oxide. Hepatology.

[48] Canbay A., Friedman S., Gores G.J. (2004). Apoptosis: The nexus of liver injury and fibrosis. Hepatology.

[49] Maher J.J. (2001). Interactions between hepatic stellate cells and the immune system. Semin. Liver Dis..

[50] Bohanon F.J., Wang X., Ding C., Ding Y., Radhakrishnan G.L., Rastellini C., Zhou J., Radhakrishnan R.S. (2014). Oridonin inhibits hepatic stellate cell proliferation and fibrogenesis. J. Surg. Res..

